# Utilizing *Andrographis paniculata* leaves and roots by effective usage of the bioactive andrographolide and its nanodelivery: investigation of antikindling and antioxidant activities through *in silico* and *in vivo* studies

**DOI:** 10.3389/fnut.2023.1185236

**Published:** 2023-05-31

**Authors:** Ramana Baru Venkata, Dintakurthi Sree Naga Bala Krishna Prasanth, Praveen Kumar Pasala, Siva Prasad Panda, Vinay Bharadwaj Tatipamula, Sirisha Mulukuri, Ravi Kumar Kota, Mithun Rudrapal, Johra Khan, Sahar Aldosari, Bader Alshehri, Saeed Banawas, Madhusudan Chetty Challa, Jithendra Kumar Kammili

**Affiliations:** ^1^Santhiram College of Pharmacy, JNTUA, Nandyal, Andhra Pradesh, India; ^2^Department of Pharmacognosy, KVSR Siddhartha College of Pharmaceutical Sciences, Vijayawada, Andhra Pradesh, India; ^3^Pharmacology Research Division, Institute of Pharmaceutical Research, GLA University, Mathura, Uttar Pradesh, India; ^4^Center for Molecular Biology, College of Medicine and Pharmacy, Duy Tan University, Da Nang, Vietnam; ^5^Department of Natural Chemistry, NGSM Institute of Pharmaceutical Sciences (NGSMIPS), Bengaluru, India; ^6^Department of Pharmaceutical Sciences, School of Biotechnology and Pharmaceutical Sciences, Vignan’s Foundation for Science, Technology & Research, Guntur, India; ^7^Department of Medical Laboratory Sciences, College of Applied Medical Sciences, Majmaah University, Al Majma’ah, Saudi Arabia; ^8^Health and Basic Sciences Research Center, Majmaah University, Al Majma’ah, Saudi Arabia; ^9^Department of Biomedical Sciences, Oregon State University, Corvallis, OR, United States

**Keywords:** andrographolide, andrographolide nanoparticles, pentylenetetrazol, antikindiling, antioxidant, network pharmacology

## Abstract

To valorise the bioactive constituents abundant in leaves and other parts of medicinal plants with the objective to minimize the plant-based wastes, this study was undertaken. The main bioactive constituent of *Andrographis paniculata,* an Asian medicinal plant, is andrographolide (AG, a diterpenoid), which has shown promising results in the treatment of neurodegenerative illnesses. Continuous electrical activity in the brain is a hallmark of the abnormal neurological conditions such as epilepsy (EY). This can lead to neurological sequelae. In this study, we used GSE28674 as a microarray expression profiling dataset to identify DEGs associated with andrographolide and those with fold changes >1 and *p*-value <0.05 GEO2R. We obtained eight DEG datasets (two up and six down). There was marked enrichment under various Kyoto Encyclopaedia of Genes and Genomes (KEGG) and Gene Ontology (GO) terms for these DEGs (DUSP10, FN1, AR, PRKCE, CA12, RBP4, GABRG2, and GABRA2). Synaptic vesicles and plasma membranes were the predominant sites of DEG expression. AG acts as an antiepileptic agent by upregulating GABA levels. The low bioavailability of AG is a significant limitation of its application. To control these limitations, andrographolide nanoparticles (AGNPs) were prepared and their neuroprotective effect against pentylenetetrazol (PTZ)-induced kindling epilepsy was investigated using network pharmacology (NP) and docking studies to evaluate the antiepileptic multi-target mechanisms of AG. Andrographolide is associated with eight targets in the treatment of epilepsy. Nicotine addiction, GABAergic synapse, and morphine addiction were mainly related to epilepsy, according to KEGG pathway enrichment analysis (*p* < 0.05). A docking study showed that andrographolide interacted with the key targets. AG regulates epilepsy and exerts its therapeutic effects by stimulating GABA production. Rats received 80 mg/kg body weight of AG and AGNP, phenytoin and PTZ (30 mg/kg i.p. injection on alternate days), brain MDA, SOD, GSH, GABAand histological changes of hippocampus and cortex were observed. PTZ injected rats showed significantly (^***^p < 0.001) increased kindling behavior, increased MDA, decreased GSH, SOD, GABA activities, compared with normal rats, while treatment AGNPs significantly reduced kindling score and reversed oxidative damage. Finally, we conclude that the leaves and roots of *A. Paniculata* can be effectively utilized for its major bioactive constituent, andrographolide as a potent anti-epileptic agent. Furthermore, the findings of novel nanotherapeutic approach claim that nano-andrographolide can be successfully in the management of kindling seizures and neurodegenerative disorders.

## Introduction

Food and medicinal plants are abundant in phytochemicals and bioactive components. Plant-based foods and medicinal products have commercial significance in agro-food, pharmaceutical and nutraceutical industries. However, the wastage of plant-based foods or bioactive substances could be attributed to be a major hurdle against the growth and sustainability of such natural resources. This issue can be resolved by maximizing the utilization of plant-based foods and medicinal constituents while minimizing their wastages. In medicinal practice, plant bioactives cannot be employed because of unsatisfactory physiological parameters or poor oral bioavailability. Therefore, the nanodelivery of bioactive components could thus maximize the medicinal/therapeutic benefits of the bioactive under investigations considering the safety and toxicity concerns. This is how biomaterials, food components and bioactive substances could have positive environmental impact through economic development and sustainability in the long run.

Medicinal plants rich in phytochemicals such as flavonoids, terpenoids, and coumarins have shown anticonvulsant activities in preclinical studies (1, 2). *Andrographis paniculata* (F. Acanthaceae) has been used in traditional medicines for curing various human ailments. The leaves of *A. paniculata* contain a diterpenoid called andrographolide (AG), which is the major bioactive component and it possesses a wide range of biological activities such as antioxidant, anti-inflammatory, neuroprotective, and anti-cancer properties. AG includes nicotine induces oxidative stress in the brain and protects against brain ischemia caused by dopamine-mediated neurotoxicity and inflammation-mediated neurodegeneration (3–5). The instability and poor water solubility of AG limits its clinical application because of its low bioavailability. Microemulsions, cyclodextrin inclusion complexes, liposomes, solid-lipid nanoparticles, niosomes etc. have been developed to enhance AG bioavailability; however, these systems have low loading capacity, poor stability and modest encapsulation efficiency (6, 7). A nanoparticle-based approach can be used to enhance the absorption, bioavailability, and biodistribution of flavonoids. In order to increase the solubility of hydrophobic drugs in water, nanoparticle drug delivery systems have been extensively used (8).

Approximating 1% of the world’s population is affected by epilepsy (EY), a complex neurological disorder, with considerable psychological, emotional and educational implications. Generally, an excessive glutamate concentration or a deficiency in GABA concentration in the central nervous system can cause a variety of pathological changes, which can be related to epilepsy (9). Several neurodegenerative conditions (for example, Alzheimer’s disease, Parkinson’s disease) are associated with oxidative stress, which causes neuronal damage. Molecular oxygen produces reactive oxygen species (ROS), which are generated by activating excitatory amino acids and releasing glutamate, causing long-term seizures and neuronal death. A direct effect of free radicals on seizures is seen when glutamate decarboxylase and glutamine synthase are deactivated, leading to a disproportionate amount of both excitatory (glutamate) and inhibitory (GABA) neurotransmitters (10–12). A scarcity of successful therapies for epilepsy exists around the world. It is possible to develop better antiepileptic treatments based on natural compounds. Patients with epilepsy may benefit from plants as a source of seizures and comorbid diseases (13).

Pentylenetetrazol kindling, a chronic epilepsy investigational model associated with seizures and neuronal plasticity, is typical in providing opportunities to study progressive behavioral variations closely resembling clinical epilepsy (14, 15). The present study was aimed to utilize andrographolide (AG) and it’s nanoformulation (AGN) as potential antikindling agent in PTZ-induced kindling rats.

## Materials and methods

### Potential targets of andrographolide

We identified the potential targets of the active compounds by analyzing the data collected from various databases. These include the TargetNet Database (TND), Comparative Toxicogenomic Database (CTD) (16), and Swiss Target Prediction (STP) (17). The chemical structures were then converted into a canonical version of SMILES using PubChem (17). The compound files were placed in the TargetNet and Swiss Target Prediction databases (16, 17). The results showed that the probability of a compound being produced was greater than 0.9. We verified that the targets were Homo sapiens using the UniProt database.

### Epilepsy related targets and shared targets of andrographolide

With the help of Geo2R, the related targets of the EY can be retrieved. The tool is part of the GEO dataset, a collection of gene expression datasets. This study aimed to identify the most common genes, which were differentially shown in different sample groups. Gene chip GSE28674 has been frequently cited in literature (18, 19). The differentially revealed genes in the samples were identified by adjusting the *p*-values to <0.05 in analyzing the data in the geo2R database using Benjamini–Hochberg method. The criteria for determining the DEGs that should be screened were FDR > 0.05 and log FC > 1 (18). Furthermore, a volcano diagram was generated using ggplot2 and using the Venn package, the tool retrieved the related targets of the EY. We also performed a comprehensive analysis of potential targets of andrographolide.

### Analysis of protein–protein interactions and hub targets

PPI analysis assists in identifying the hub targets related to AG on EY. The PPI network was constructed by using STRING with the “Homo sapiens” setting to retrieve the shared targets of andrographolide with ER (20). The network properties were analyzed using Cytoscape 3.7.2 software by selecting the “Analysis Network” function. The degree of freedom (DOF) plays an influential role in a PPI network because points overhead the average DOF typically play a significant role (21).

### Kyoto Encyclopaedia of Genes and Genomes analysis and Gene Ontology enrichment

KEGG pathway enrichment analysis and GO function enrichment analysis were performed using DAVID with Homo sapiens as the selected species. Visualization was performed using the online tool Weishengxin (2). A threshold level of *p* < 0.05 is used for all GO enrichment and pathway analyses. The final pathway map was created by combining pathways with the highest scores.

### Network construction

By using Cytoscape 3.7.2 software, a primary regulatory network was used to construct in order to visualize the “drug–target–disease” correlation between AG and EY. The nodes in this network are the shared targets, active compounds, and pathways. The edges show how compounds, targets, and pathways interact.

### Molecular docking

Andrographolide was retrieved from the 3D.sdf file of the PubChem database. Using OpenBabel-2.3, the SDF files were converted to pdb files, which were then converted into pdbqt files using AutoDock Tools (version 1.5.6) (22–24). The x-ray crystal structure of the gamma-aminobutyric acid receptor GABA(A)R-beta3 (PDB ID 4COF) (25–33) was obtained from the RCSB Protein Data Bank. MGL AutoDock Tools were used to prepare the protein, including removing crystal water and ligands and adding Kollman charges and polar hydrogen atoms. The PDB structured proteins were transformed to the pdbqt format utilizing Autodock. Ten distinct poses of the ligand molecules were acquired after docking with target proteins (*x*, *y*, *z* = 34.55, 56.37, 23.86) by employing AutoDock Vina’s standard settings (34–39). The Biovia Discovery Studio was chosen to illustrate ligand-protein interactions (40–47).

### Preparation of andrographolide nanoparticles

An antisolvent (n-hexane) was added to absolute ethanol (15 mg/mL) to obtain the nanosuspension of andrographolide. The n-hexane to ethanol ratio was 10:1 to facilitate the process of nanosuspension. Subsequently, the nanosuspension of the medication was placed into a round-bottom flask and rotated at 90 rpm at a temperature of 40°C and a pressure of 300 mbar, and the solvent was then removed using a rotary evaporator. Next, the solid in the flask was evaporated and dried (48). The particle sizes of the prepared AGN and AG particles were measured at an angle of 900° using the dynamic light scattering technique.

### Animals

The albino Wistar male rats were weighed between 150–200 grams, and they were held in a room with a temperature of 25°C, 55% relative humidity, and a 12/12 h light/dark cycle, which complies with CPCSEA regulations. Experimental rats were fed with pellet diet, and water *ad libitum*. The IAEC (1725/GO/a/13/CPCSEA) evaluated and accepted the current experimental protocol.

### Treatment protocol

#### Kindling induction

Rats were treated with sub-convulsant dose of PTZ (35 mg/kg/b.wt. i.p./alternative days) until kindling was developed. The Racine scale was used to monitor the intensity of seizures was monitored for around 30 min following each injection (49). Kindling intensity is monitor based on the following score:

Score 0 = No response,

Score = 1 facial and mouth jerks,

Score = 2 myoclonic body jerksor nodding,

Score = 3 forelimb clonus, rearing, hindlimb clonus, falling down and forelimbtonus.

Score = 4 tonic extension of the hindlimb,

Score = 5 status epilepticus and/or death.

In the study, rats with convulsions on the first day were excluded. When rats exhibit stage 5 seizures, they are considered fully kindled. The doses (50), phenytoin (51), and andrographolide (52) have been determined in previous studies.

Rats were divided in to five groups, each group contain six animals.

Group I: Received vehicle saline i.p.

Group II: PTZ (35 mg/kg/b.wt. i.p./alternative days).

Group III: Phenytoin (35 mg/kg/b.wt. i.p./daily) + PTZ (35 mg/kg/b.wt. i.p./alternative days) treatment.

Group IV: Phenytoin (35 mg/kg/b.wt. i.p.) + AG (80 mg/kg. p.o) + PTZ (35 mg/kg/b.wt. i.p./alternative days) treatment.

Group V: PTZ + phenytoin (35 mg/kg/b.wt. i.p.) + AGN (80 mg/kg.p./o) + PTZ (35 mg/kg/b.wt. i.p./alternative days).

The ends of the treated rats were executed under ether anesthesia, and the brains were quickly removed, cleaned with ice-cold saline, and subjected to estimation of malonaldehyde (MDA), reduced glutathione (GSH) (53), superoxide dismutase (SOD) (54), and gamma-aminobutyric acid (GABA) (53).

### Histopathological examination

Toluidine blue and hematoxylin and eosin were used for staining the sections (55). The hippocampal and cortical regions of the slides were photographed digitally under a microscope.

### Statistical analysis

The mean value and standard error of the mean (SEM) for data gathered from three trials is presented. A significant difference (*p* < 0.001) was identified between the kindling rats and treatment rats when using one-way ANOVA followed by Dunnett’s comparison test.

## Results

### Screening of potential targets in andrographolide

This study identified 423 targets of andrographolide. It is possible that andrographolide may have similar biological effects as andrographide and that when combined, these effects may be synergistic. The integration of all targets resulted in 371 targets corresponding to andrographolide.

### Related targets of andrographolide and shared targets of andrographolide against epilepsy

**Figure 1:**
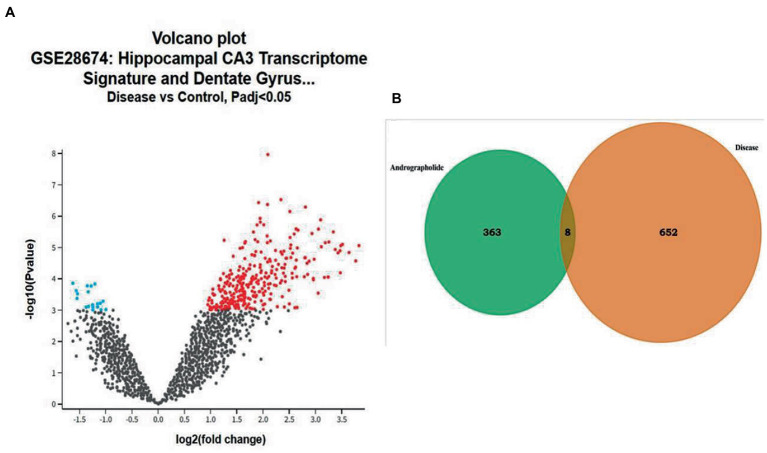
Genes with differential expression in epilepsy (EY). **(A)** A red or blue gene represents an upregulated gene, whereas a blue gene represents a downregulated gene, based on the standard FDR of 0.05 and log FC > 1. **(B)** Potential targets of andrographolide and differentially expressed genes in EY.

Gene chip GSE28674 was used to examine the impact of dentate gyrus MRI features and hippocampal CA3 transcriptome signature in relation to initial precipitating injury in refractory temporal lobe epilepsy, including twelve normal samples (hippocampus no febrile seizures) and six samples (hippocampus febrile seizures). As shown in Figure 1A, 660 DEGs were identified between normal samples and disease samples using the above screening criteria of FDR > 0.05 and log FC > 1. To further understand andrographolide’s mechanism of action in treating EY, a Venn diagram (Figure 1B) was used to identify eight shared targets (Table 1) between 660 DEGs and 371 potential targets.

**Table 1:** The eight shared targets between the andrographolide and epilepsy with FDR < 0.05 and log FC > 1.

Gene	log FC	FDR
DUSP10	−1.00026	0.010221
FN1	1.571008	0.014388
AR	−1.1762	0.010014
PRKCE	1.284686	0.045194
CA12	1.315168	0.026297
RBP4	2.214523	0.020294
GABRG2	2.807807	0.004511
GABRA2	1.089175	0.014506

### Kyoto Encyclopaedia of Genes and Genomes pathway enrichment and Gene Ontology functional annotation analysis

**Figure 2:**
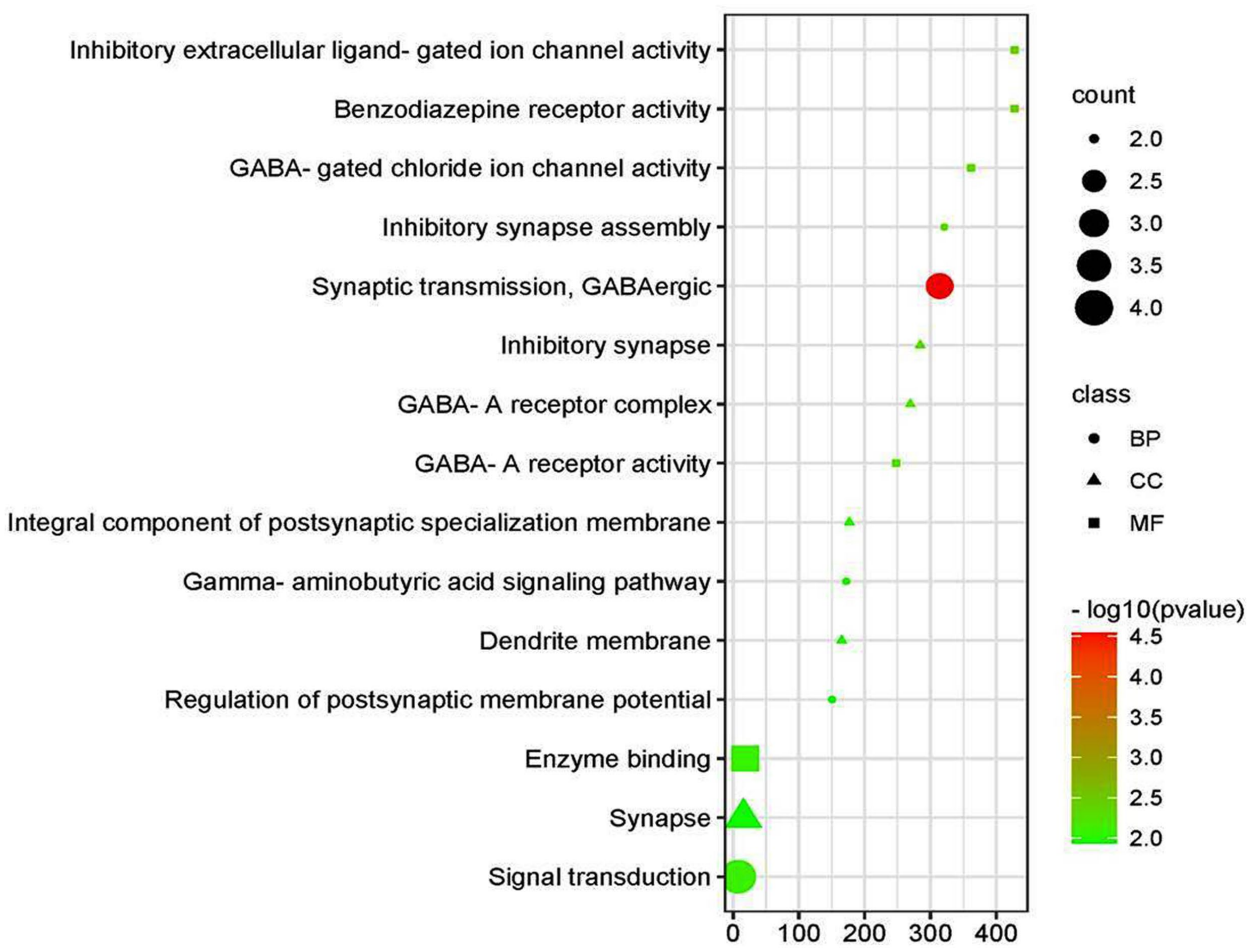
Bubble chart showing the relationship between KEGG pathways and associated genes.

Based on the GO functional annotation analysis, 11 CC terms, 16 BP terms, and 10 MF terms predominated across the eight shared targets. As shown in Figure 2 and Table 2, the top five enrichment results are presented for each part. Synaptic transmission, GABAergic inhibitory synapse assembly, GABA signaling pathway, signal transduction, and regulation of postsynaptic membrane potential are closely connected to BP. Concerning CC, higher enrichment was found in the synapse, dendrite membrane (GABA-A receptor complex), postsynaptic specialization membrane, and plasma membrane. The main terms of EY in MF included benzodiazepine receptor activity, GABA-gated chloride ion channel activity, GABA-A receptor activity, inhibitory extracellular ligand-gated ion channel activity, and enzyme binding activities. The current analysis exhibited that these targets were closely associated to the activation of GABA ion channels, causing an influx of chloride ions and leading to hyperpolarization and decreased excitability.

**Table 2:** Gene Ontology (GO) enrichment analysis performed on eight common genes.

Term	Description	Count	*p*-value	Genes	Fold enrichment	Bonferroni
Biological process						
GO:0051932	Synaptic transmission, GABAergic	3	2.85549E−05	GABRA2, PRKCE, GABRG2	313.9565217	0.005126771
GO:1904862	Inhibitory synapse assembly	2	0.005440965	GABRA2, GABRG2	320.9333333	0.625456681
GO:0007165	Signal transduction	4	0.008137434	GABRA2, AR, PRKCE, GABRG2	7.599052881	0.770242346
GO:0007214	GABA signalling pathway	2	0.010135923	GABRA2, GABRG2	171.9285714	0.84019093
GO:0060078	Regulation of postsynaptic membrane potential	2	0.011576698	GABRA2, GABRG2	150.4375	0.877048021
Cellular components						
GO:0060077	Inhibitory synapse	2	0.006139434	GABRA2, GABRG2	284.3333333	0.242041103
GO:1902711	GABA-A receptor complex	2	0.006479565	GABRA2, GABRG2	269.3684211	0.253626525
GO:0099060	Integral component of postsynaptic specialization membrane	2	0.009875383	GABRA2, GABRG2	176.4827586	0.360200909
GO:0032590	Dendrite membrane	2	0.010553352	GABRA2, GABRG2	165.0967742	0.379620876
GO:0045202	Synapse	3	0.010999223	GABRA2, PRKCE, GABRG2	15.73155738	0.392077144
Molecular function						
GO:0008503	Benzodiazepine receptor activity	2	0.004086826	GABRA2, GABRG2	427.5227273	0.255360015
GO:0005237	Inhibitory extracellular ligand-gated ion channel activity	2	0.004086826	GABRA2, GABRG2	427.5227273	0.255360015
GO:0022851	GABA-gated chloride ion channel activity	2	0.004828345	GABRA2, GABRG2	361.75	0.294241982
GO:0004890	GABA-A receptor activity	2	0.007050064	GABRA2, GABRG2	247.5131579	0.399145455
GO:0019899	Enzyme binding	3	0.008403619	AR, PRKCE, FN1	18.0875	0.455352909

GO analysis showed 16 entries for biological processes, 11 for cell components, and 10 for molecular functions (*p* < 0.05). The top five entries with the most significant *p*-values are shown.

**Figure 3:**
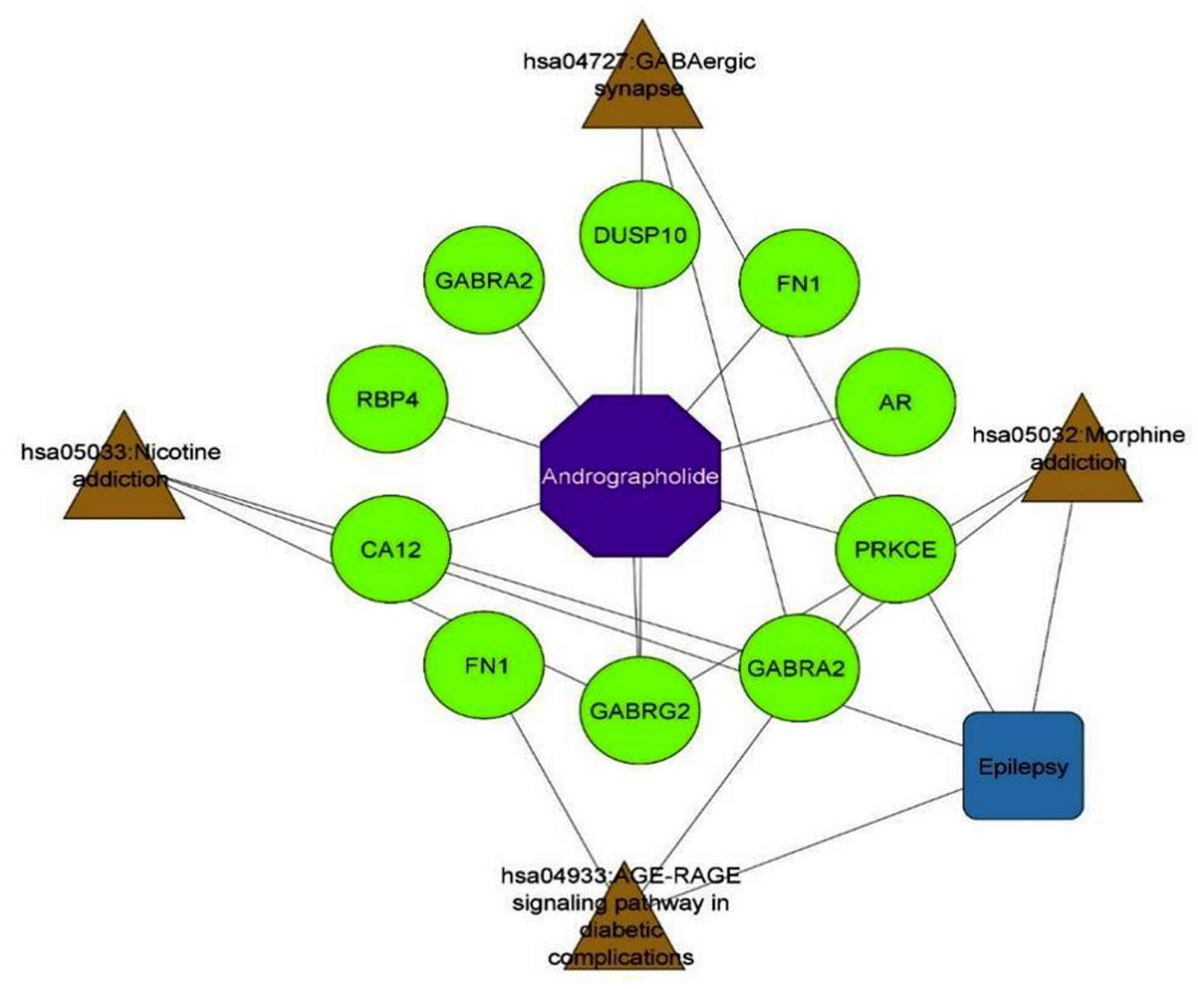
Component-target-signal pathway network.

**Figure 4:**
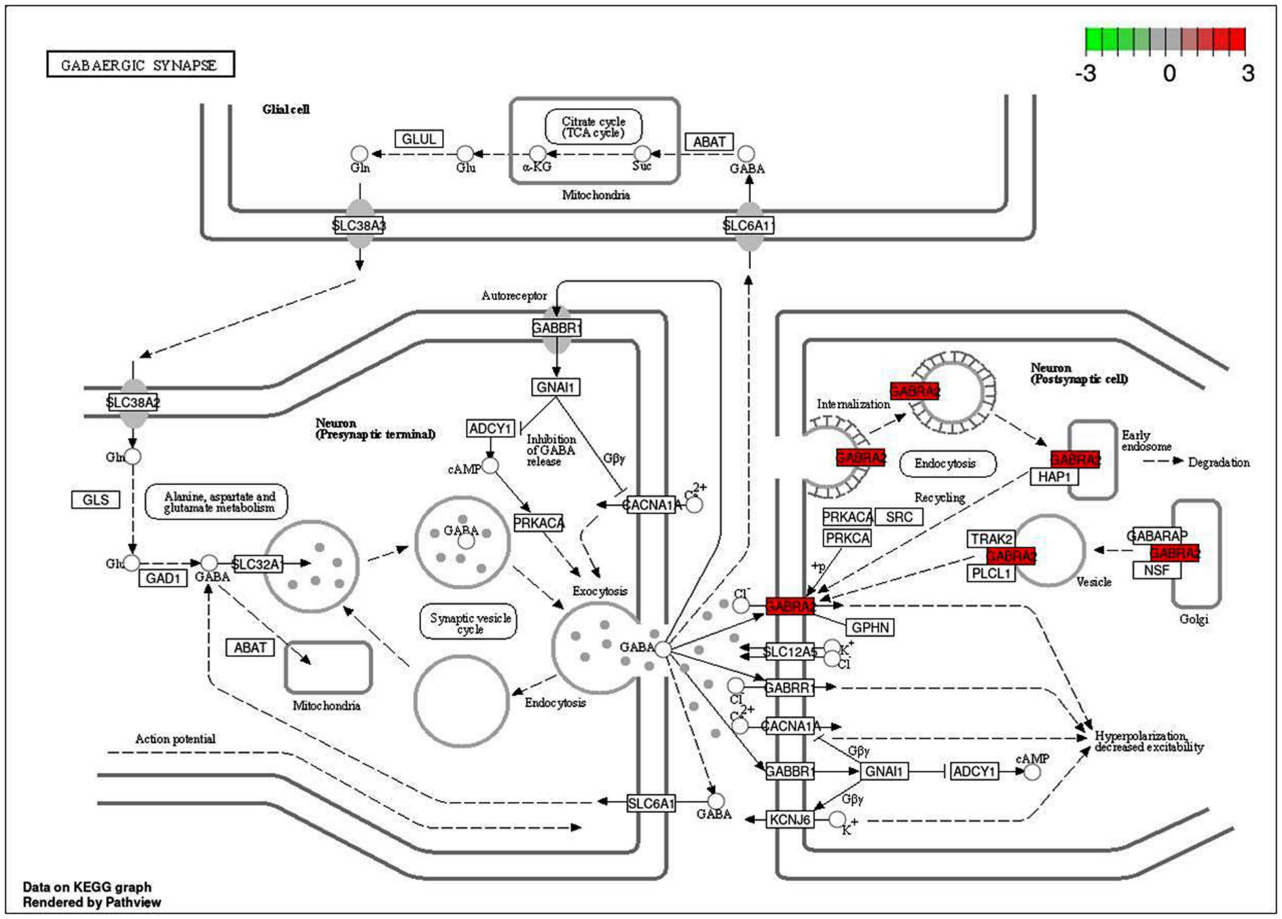
Mechanisms of action of andrographolide against EY.

An intensive KEGG enrichment analysis was conducted on the eight shared targets, targeting the anti-EY pathway of andrographolide, and results showed an FDR of <0.05. The outcomes are presented in Figure 3. The four enriched pathways were GABAergic synapse (hsa04727), nicotine addiction (hsa05033), morphine addiction (hsa05032), and the AGE-RAGE signaling pathway in diabetic complications (hsa04933) (Table 3). For instance, GABRA2 and GABRG2 appear in three pathways, indicating that andrographolide mainly acts by activating GABA ion channel receptors (Figure 4).

**Table 3:** Target genes in 4 signaling pathways enrichment related to EY.

Description	Count	*p*-value	Genes	Fold enrichment	Bonferroni
hsa05033:Nicotine addiction	2	0.029076553	GABRA2, GABRG2	58.25714286	0.654331654
hsa04727:GABAergic synapse	2	0.033731908	GABRA2, GABRG2	26.18298555	0.906586848
hsa05032:Morphine addiction	2	0.045124214	GABRA2, GABRG2	25.60753532	0.911459737
hsa04933:AGE-RAGE signaling pathway in diabetic complications	2	0.05136826	PRKCE, FN1	23.30285714	0.930438835

**Figure 5:**
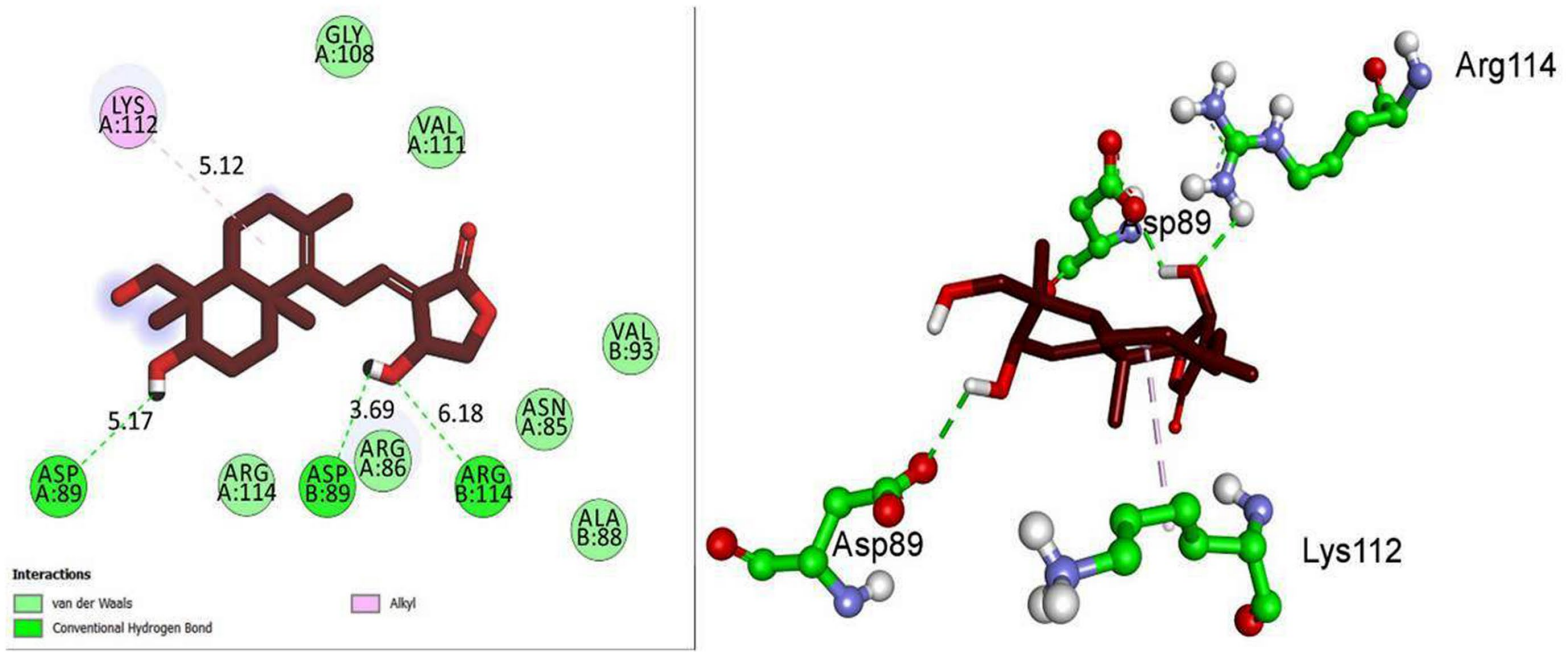
Molecular docking interactions of andrographolide in 2D and 3D representations.

An enrichment analysis of andrographolide’s anti-EY pathway was conducted with KEGG, with FDR < 0.05. Figure 5 illustrates the results. The four enriched pathways were GABAergic synapse (hsa04727), nicotine addiction (hsa05033), morphine addiction (hsa05032), and the AGE-RAGE signaling pathway in diabetic complications (hsa04933). For instance, GABRA2 and GABRG2 appear in three pathways, indicating that andrographolide mainly acts by activating GABA ion channel receptors.

### Molecular docking

Based on KEGG enrichment analysis, GABAergic synapse signaling pathways appear to be a hub signaling pathway of andrographolide against EY. The GABRA2 and GABRG2 genes were related to three signaling pathways, including hsa05033, nicotine addiction; hsa04727, GABAergic synapse; and hsa05032, morphine addiction. Therefore, GABA is considered to be a prominent target of andrographolide in the treatment of epilepsy.

Based on molecular docking studies, andrographolide binds to the GABA receptor with a binding energy of −6.8 kcal/mol. It formed three hydrogen bonds with ASP A:89, ASP B:89, and ARG B:114, with distances of 5.17, 3.69, and 6.18 A°. It also interacted with LYS A:112 *via* hydrophobic interactions at a distance of 5.12 A° (Figure 5). This suggests that andrographolide may be an important ligand for controlling glucose homeostasis.

### Particle size and charge of andrographolide nanoparticles

**Figure 6:**
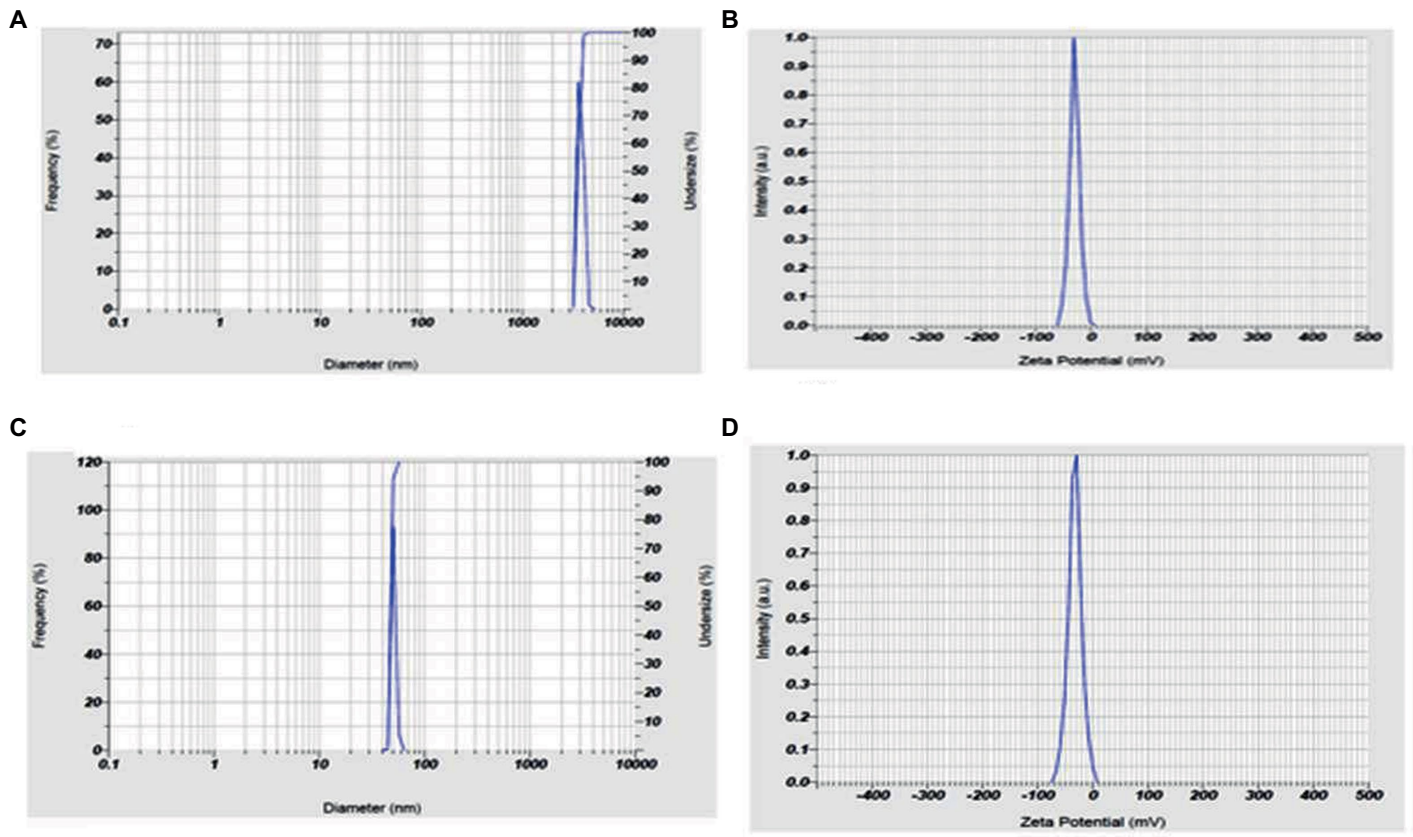
**(A)** Andrographolides particle size, **(B)** andrographolides particle charge, **(C)** andrographolides nanoparticle size, **(D)** andrographolides nanoparticle charge.

The andrographolide particles were found to have a size of 3587.8 nm (3.6 microns), indicating their large particle nature. On the other hand, the size of the prepared nano-andrographolide was found to be 47.9 nm indicating that the prepared nanoparticles were of good size. The nano-andrographolide particles exhibited −32.3 mV charge indicating that the particles remained stable and free of agglomeration (Figure 6).

### Effect of andrographolide nanoparticle treatment on pentylenetetrazol-induced kindling model

After three injections, PTZ-treated rats reached seizure severity stage 5 and died. In contrast, the phenytoin +AG combination did not cause severe seizures in rats. Up to the ninth PTZ injection, AGN + phenytoin combinations were seizure-free, but the severity of the seizures increased following the eleventh PTZ injection. Our results agree with those of various studies in which PTZ was frequently used to induce seizure severity (56–58). In this study, the number of animals that developed kindling significantly decreased after AGN treatment (Tables 4–7).

**Table 4:** Effect of PTZ (35 mg/kg).

Days	PTZ inj.		Score 1 (time)	Score 2 (time)	Score 3 (time)	Score 4 (time)	Score 5 (time)
1	1	OA	4 min	NO	NO	NO	NO
		DA	8 min	NO	NO	NO	NO
3	2	OA	NO	NO	3 min	NO	NO
		DA	NO	NO	12 min	NO	NO
5	3	OA	NO	NO	NO	NO	3 min followed by death (100%)
		DA	NO	NO	NO	NO	

**Table 5:** Effect of PTZ (35 mg/kg) + phenytoin (35 mg/kg).

Days	PTZ inj.		Score 1 (time)	Score 2 (time)	Score 3 (time)	Score 4 (time)	Score 5 (time)
1	1	OA	NO	NO	NO	NO	NO
		DA	NO	NO	NO	NO	NO
3	2	OA	3 min	NO	NO	NO	NO
		DA	9 min	NO	NO	NO	NO
5	3	OA	NO	NO	5 min	NO	NO
		DA	NO	NO	13 min	NO	NO
7	4	OA	NO	NO	NO	9 min	NO
		DA	NO	NO	NO	15 min	NO
9	5	OA	NO	NO	NO	NO	5 min followed death (83.3%)
		DA	NO	NO	NO	NO	

**Table 6:** Andrographolide (50 mg/kg) + PTZ (35 mg/kg).

Days	PTZ inj.		Score 1 (Time)	Score 2 (Time)	Score 3 (Time)	Score 4 (Time)	Score 5 (Time)
1	1	OA	5 min	NO	NO	NO	NO
		DA	8 min	NO	NO	NO	NO
3	2	OA	4 min	NO	NO	NO	NO
		DA	11 min	NO	NO	NO	NO
5	3	OA	NO	3 min	NO	NO	NO
		DA	NO	15 min	NO	NO	NO
7	4	OA	NO	NO	3 min	NO	NO
		DA	NO	NO	20 min	NO	NO
9	5	OA	NO	NO	NO	NO	10 min followed death (66.6%)
		DA	NO	NO	NO	NO	

**Table 7:** Andrographolide nanoparticles (50 mg/kg) + PTZ (35 mg/kg).

Days	PTZ inj.		Score 1 (time)	Score 2 (time)	Score 3 (time)	Score 4 (time)	Score 5 (time)
1	1	OA	6 min	NO	NO	NO	NO
		DA	10 min	NO	NO	NO	NO
3	2	OA	5 min	NO	NO	NO	NO
		DA	11 min	NO	NO	NO	NO
5	3	OA	NO	5 min	NO	NO	NO
		DA	NO	12 min	NO	NO	NO
7	4	OA	NO	4 min	NO	NO	NO
		DA	NO	15 min	NO	NO	NO
9	5	OA	NO	NO	4 min	NO	NO
		DA	NO	NO	16 min	NO	NO
11	6	OA	NO	NO	8 min	NO	NO
		DA	NO	NO	20 min	NO	NO
13	7	OA	NO	NO	NO	15 min	NO
		DA	NO	NO	NO	5 min	NO
15	8	OA	NO	NO	NO	20 min	NO
		DA	NO	NO	NO	7 min	NO
17	9	OA	NO	NO	NO	10 min	NO
		DA	NO	NO	NO	9 min	NO
19	10	OA	NO	NO	NO	NO	15 min
		DA	NO	NO	NO	NO	5 min
21	11	OA	NO	NO	NO	NO	20 min
		DA	NO	NO	NO	NO	7 min

NO, not observed; OA, onset of action; DA, duration of action.

### Effect of andrographolides nanoparticles on pentylenetetrazol kindling-induced brain oxidative biomarkers

**Figure 7:**
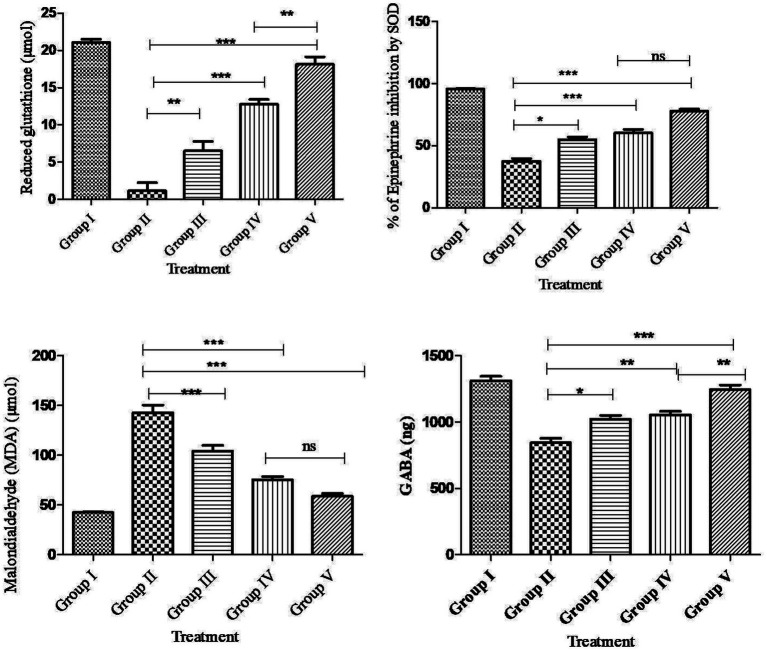
Brain biochemical parameters. *p*<0.001***.

Compared to the normal control rats, PTZ treatment had a significant (*p* < 0.001^***^) decrease in SOD, GSH, GABA and an increase in MDA levels in the rat brain. Compared to PTZ alone treatment and AG treatment, AGN treatment had the effect of significantly (*p* < 0.001^***^) increasing SOD, GSH, GABA, and reducing MDA levels in the brain (Figure 7).

PTZ selectively blocks the chloride ionophore complex of the GABA receptor. Following repeated or single administration, its convulsant effects affect GABAergic, adenosinergic, and glutamatergic systems. In this study, PTZ treatment significantly decreased GABA activity, whereas the combination of AGN with phenytoin increased GABA activity compared with AG with phenytoin and phenytoin treatment. In this context, PTZ has also been shown to activate nucleases, phospholipases, and membrane proteases that result in the degradation of cytoskeletal proteins, membrane phospholipids, and protein phosphorylation (58–60). After PTZ-induced seizures, significant reductionin GSH and SOD activity (61) and increased MDA activity (62) have been observed in animal brain homogenates. Reactive oxygen species are produced in an unreliable manner as a result of antiepileptic medications including phenytoin, valproic acid, and carbamazepine (63). Epilepsy and associated neurological comorbidities can be improved when these drugs are combined with antioxidants (64, 65). An examination of histology showed a decrease in cell count and cell death in the cortex, CA1 and CA3 region of the rat’s hippocampus. The group receiving PTZ showed a significant increase in dead cells and a decreased density of cells compared with the control value. Supplementary Figures S1–S3 showed that the phenytoin + AGN + PTZ group had significantly fewer dead cells and greater cell density in the hippocampus and cortex in comparison with the AGN group. However, the combination of AGN and phenytoin treatment restored cellular antioxidant enzymes when compared with the combination of AG and phenytoin treatment in the PTZ group.

## Conclusion

Based on the network pharmacology analysis, the andrographolide acts as an anti-epileptic agent by upregulating the GABA levels, and further molecular docking studies confirmed the same. PTZ administration revealed kindling development, greater oxidative stress, diminished antioxidant activity, augmented GABA levels, and neurodegeneration. However, the combination of phenytoin and andrographolide nanoparticles significantly reduced the seizure score (% of kindled animals). The above findings indicate that the potential anti-kindling effects of andrographolide nanoparticles may protect against oxidative stress and increase GABAergic activity in kindling seizures and thereby modulates neuroprotection in the cortex. Finally, we conclude that the leaves and roots of *A. paniculata* can be effectively utilized for its major bioactive constituent, andrographolide as a potent anti-epileptic agent. Furthermore, the findings of novel nanotherapeutic approach claim that nano-andrographolide can be successfully in the management of kindling seizures and neurodegenerative disorders.

## Data availability statement

The original contributions presented in the study are included in the article/Supplementary material, further inquiries can be directed to the corresponding authors.

## Ethics statement

The animal study was reviewed and approved by Santhiram College of Pharmacy, JNTUA, Nandyal, Andhra Pradesh, India.

## Author contributions

RB, DP, PP, SP, and VT: conceptualization, methodology, and software. SM, RK, MR, and JK: investigation, writing—original draft, review, and editing. PP, RK, MC, JKK, SA, BA, and SB: resources and supervision, validation, and formal analysis. JK, SA, BA, and SB: funding acquisition. SM and MR: critical analysis, final draft-review, and editing. All authors contributed to the article and approved the submitted version.

## Conflict of interest

The authors declare that the research was conducted in the absence of any commercial or financial relationships that could be construed as a potential conflict of interest.

## Publisher’s note

All claims expressed in this article are solely those of the authors and do not necessarily represent those of their affiliated organizations, or those of the publisher, the editors and the reviewers. Any product that may be evaluated in this article, or claim that may be made by its manufacturer, is not guaranteed or endorsed by the publisher.
