# Supplementary material for: Utilizing Andrographis paniculata leaves and roots by effective usage of the bioactive andrographolide and its nanodelivery: investigation of antikindling and antioxidant activities through in silico and in vivo studies
Source: Front Nutr. 2023 May 31;10:1185236. doi: 10.3389/fnut.2023.1185236 (PMC10266967; doi:10.3389/fnut.2023.1185236)
Supplement: Supplementary file 1 [file Data_Sheet_1.docx]

**Supplementary file**


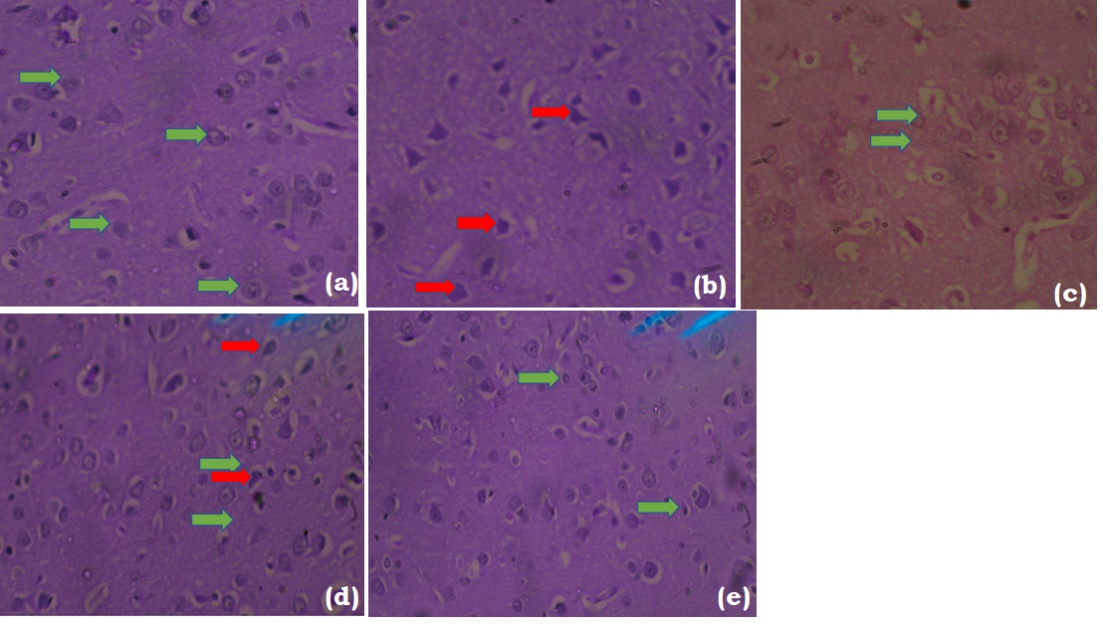


**Fig. S1.** Cortex histopathology (Magnification 400X) [Green arrow indicating- cell with Nucleus, Red arrow indicating – Dead cells]. a) Group I, b) Group, II c) Group III, d) Group IV, e) Group V


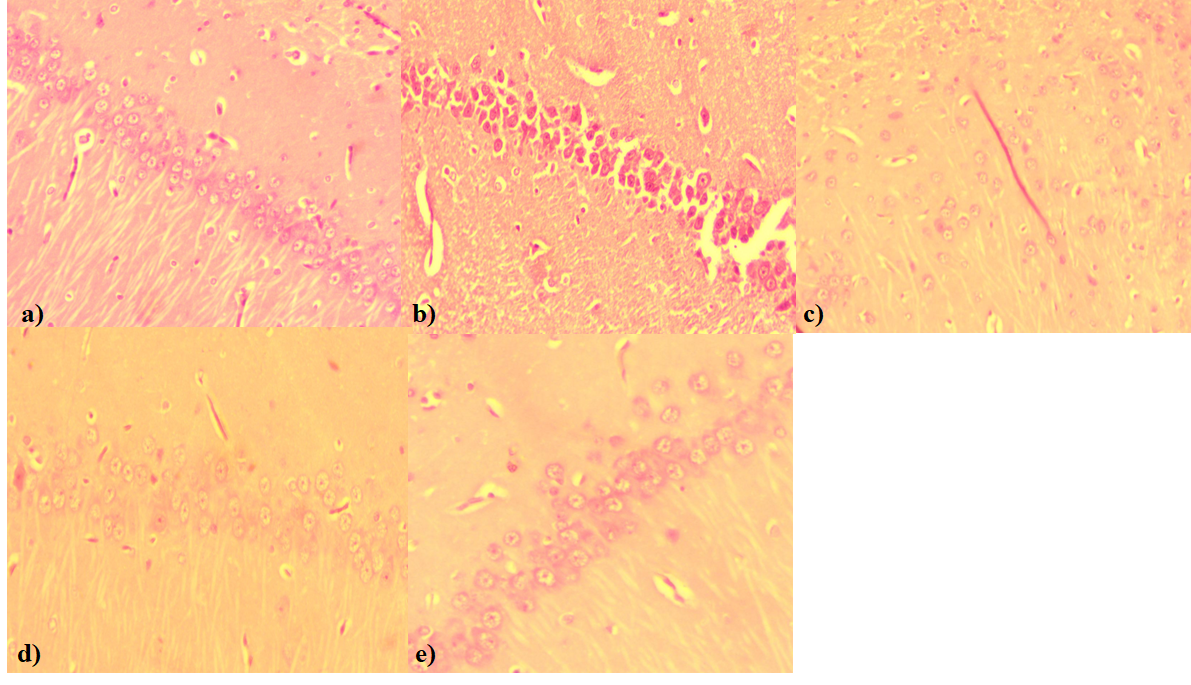


**Fig. S2.** CA 1 Hippocampus histopathology (Magnification 400X). a) Group I, b) Group, II c) Group III, d) Group IV, e) Group V


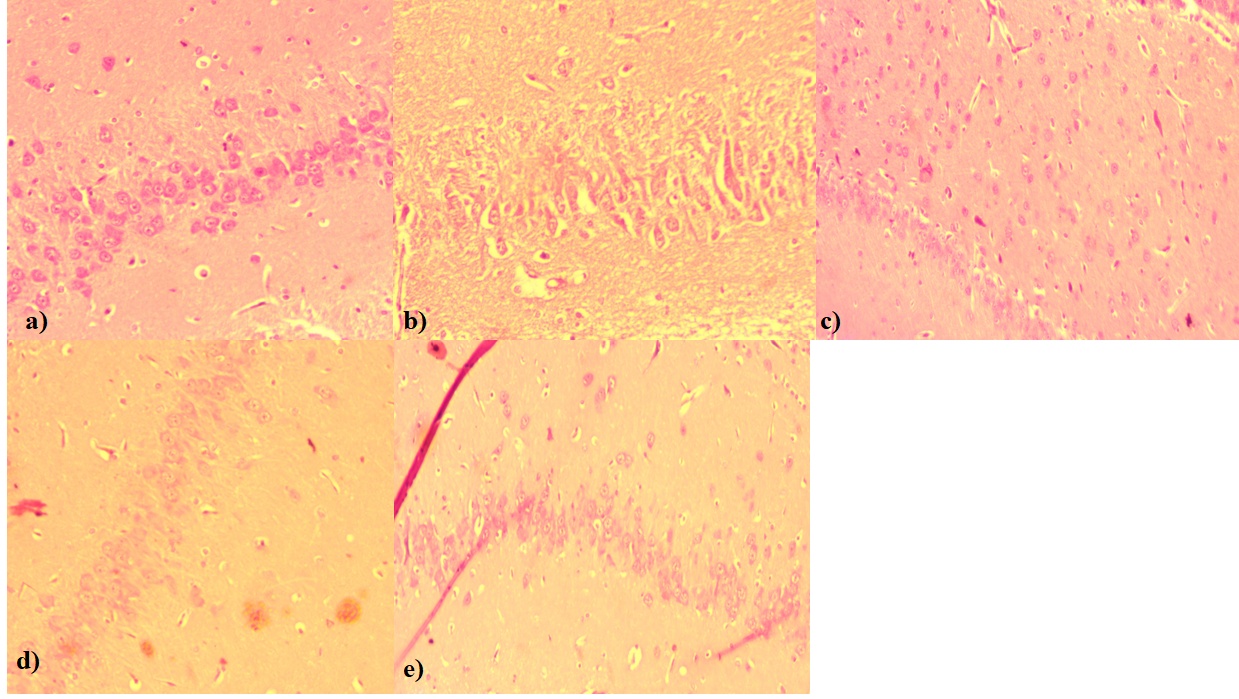


**Fig. S3.** CA 3 Hippocampus histopathology (Magnification 400X). a) Group I, b) Group II, c) Group III, d) Group IV, e) Group V
